# The role of scientific evidence in decisions to adopt complex innovations in cancer care settings: a multiple case study in Nova Scotia, Canada

**DOI:** 10.1186/s13012-019-0859-5

**Published:** 2019-02-12

**Authors:** R. Urquhart, C. Kendell, L. Geldenhuys, A. Ross, M. Rajaraman, A. Folkes, L. L. Madden, V. Sullivan, D. Rayson, G. A. Porter

**Affiliations:** 1 Department of Surgery, Dalhousie University, Room 8-032, Centennial Building, 1276 South Park Street, Halifax, Nova Scotia B3H 2Y9 Canada; 2 Nova Scotia Health Authority, Halifax, Nova Scotia Canada; 3 Department of Community Health and Epidemiology, Dalhousie University, Halifax, Nova Scotia Canada; 4 Department of Pathology, Dalhousie University, Halifax, Nova Scotia Canada; 5 Department of Radiology, Dalhousie University, Halifax, Nova Scotia Canada; 6 Department of Radiation Oncology, Dalhousie University, Halifax, Nova Scotia Canada; 7 Department of Medical Oncology, Dalhousie University, Halifax, Nova Scotia Canada

**Keywords:** Adoption, Innovation, Evidence, Case study methods

## Abstract

**Background:**

Health care delivery and outcomes can be improved by using innovations (i.e., new ideas, technologies, and practices) supported by scientific evidence. However, scientific evidence may not be the foremost factor in adoption decisions and is rarely sufficient. The objective of this study was to examine the role of scientific evidence in decisions to adopt complex innovations in cancer care.

**Methods:**

Using an explanatory, multiple case study design, we examined the adoption of complex innovations in five purposively sampled cases in Nova Scotia, Canada. Data were collected via documents and key informant interviews. Data analysis involved an in-depth analysis of each case, followed by a cross-case analysis to develop theoretically informed, generalizable knowledge on the role of scientific evidence in innovation adoption that may be applied to similar settings and contexts.

**Results:**

The analyses identified key concepts alongside important caveats and considerations. Key concepts were (1) scientific evidence underpinned the adoption process, (2) evidence from multiple sources informed decision-making, (3) decision-makers considered three key issues when making decisions, and (4) champions were essential to eventual adoption. Caveats and considerations related to the presence of urgent problems and short-term financial pressures and minimizing risk.

**Conclusions:**

The findings revealed the different types of issues decision-makers consider while making these decisions and why different sources of evidence are needed in these processes. Future research should examine how different types of evidence are legitimized and why some types are prioritized over others.

**Electronic supplementary material:**

The online version of this article (10.1186/s13012-019-0859-5) contains supplementary material, which is available to authorized users.

Contributions to the literature

- Research has shown that the scientific evidence does not always have a large influence on decisions to adopt innovations in health care. For many decision-makers, experiential knowledge can be more relevant and applicable.
- Although we found scientific evidence typically underpinned the adoption process, the types of evidence most valued by strategic-level decision-makers were insights into real-world implementation challenges and impact obtained from other jurisdictions.
- These findings contribute to recognized gaps in the literature, including ascertaining how, when, and why different types of evidence are used during decisions to adopt innovations in health care.

## Background

Health care delivery and outcomes can be improved by using innovations (i.e., new ideas, technologies, and practices [1]) supported by scientific evidence [2, 3]. Nevertheless, well-documented evidence-practice gaps exist across healthcare settings, conditions, and jurisdictions [4–6]. Adoption and implementation of innovations in health care are complex and dynamic processes, requiring strong evidentiary support [7]. Nevertheless, scientific evidence may not be the foremost factor in adoption decisions, and by itself is rarely sufficient [8]. The nature of evidence for health care improvement can be ambiguous and understandings of what constitutes sufficient and appropriate evidence (e.g., scientific evidence, clinical/professional experience, local data, patient values/preferences) differ across professional groups [9–11]. Moreover, when making decisions about innovations, scientific evidence will have to be interpreted alongside local resources and constraints and clinical or policy priorities.

How evidence is identified and the role each type plays when individuals and teams decide to adopt innovations is often unclear [7, 12, 13]. A systematic review on the barriers to and facilitators of the use of evidence in policy decisions found it was difficult to ascertain the role of scientific evidence and other factors (e.g., resource constraints, costs, socio-political environment) influencing policy processes, partly because most researchers neither define what they mean by evidence nor explore *how* and *why* different factors come into play during the policymaking process [14]. The authors highlighted the need for empirical studies using novel methodologies to permit the identification and exploration of decision-making processes and how scientific evidence influences policy alongside other important factors, such as resources and the socio-political environment. Similarly, recent reviews emphasized the need for qualitative research to understand how and why different types of evidence are used during decision-making processes [15, 16].

The objective of this study was to examine the role of scientific evidence in decisions to adopt complex innovations in cancer care, including (a) how scientific evidence is considered alongside other sources of evidence (e.g., clinical experience, knowledge from patients and caregivers, and local data), (b) whether and how the role of scientific evidence differs across types of innovations, and (c) how the use of scientific evidence is influenced by contextual conditions. By *scientific evidence*, we refer to evidence available from health research—i.e., evidence produced through a process that involves testing of a formalized theory or hypothesis, use of recognized and replicable methods to collect data, and use of recognized and replicable methods to analyze and interpret the data [17]. By *adoption*, we refer to “a decision to make full use of an innovation as the best course of action available” [18]. By *complex innovation*, we refer to an idea, technology, or practice that an organization is using for the first time and that requires active, coordinated actions by many individuals and/or professional groups to achieve benefits [1].

## Methods

Using case study methodology [19], an explanatory, multiple case design was selected to explain *how* decision-making unfolded and interpret the data on a comprehensive theoretical level. This study was informed by the Consolidated Framework for Implementation Research (CFIR) [20].

### Case sampling

In case study research, strategic case sampling is paramount to optimizing the applicability and relevance of findings to different settings [21]. The careful selection of cases provides the opportunity to examine processes that are central to our understanding of new or existing theory related to the phenomenon in question [22]. Five cases in Nova Scotia, Canada, were selected for study (Table 1). Nova Scotia has a population of approximately 940,000. Cases were sampled to obtain variation on three criteria: (1) type of innovation, (2) evidentiary base, and (3) contextual factors (e.g., setting, timing, individuals involved). A two-phased approach was used to identify and select cases that would meet the sampling criteria and thus optimize transferability across types of innovations and contexts. First, the lead researcher [RU] met individually with clinical and administrative stakeholders to develop an inventory of potential cases. Second, an in-person meeting was held among research team members and key stakeholders to discuss the inventory of potential cases and select the final cases for study. Emphasis was placed on selecting the most appropriate and information-rich cases. Five cases were selected to achieve diversity on key elements (i.e., sampling criteria), to increase the transferability of findings, and to maintain a reasonable number of cases so the cases could be studied in sufficient detail and depth. All cases had been adopted into the cancer care system. Following the in-person meeting, the research team further delineated the boundaries of each case, including the time period of study and the types of evidence to be collected. This sampling approach was intended to optimize the applicability of the study’s findings by ensuring that cases were carefully selected, ranging from typical to deviant [21], and that potentially strategic cases were not overlooked.

**Table 1:** Key elements of each case

	Innovation description	Main sources of evidence	Key resources and activities required for implementation	Decision process/length
Case 1: PET	Nuclear medical imaging technology, often combined with CT imaging, to provide additional functional imaging detail Supported by scientific evidence for better cancer diagnosis, staging, and/or response to therapy for certain cancer types	Scientific evidence Patient experience	• Capital equipment purchase • Access to isotopes* • Expertise in PET scanning • Policy pertaining to use (only to be used for certain indications)	Formal requests/proposals to successive levels of system, ending with government**; required approval at all levels Decision process lasted approx. 8 years with adoption occurring in 2008
Case 2: IMRT	Type of radiotherapy that delivers targeted radiation to tumors, with better sparing of surrounding normal tissue Supported by scientific evidence for certain cancer types and indications	Scientific evidence Clinical experience Local data Data from other jurisdictions	• Integration with existing imaging modalities • Policy pertaining to use (only to be used for certain indications) • Education/training for all members of multi-disciplinary team	No formal request; informally adopted at departmental level Decision process lasted approx. 2 years with adoption occurring in 2005
Case 3: MSI testing	Molecular biology technique to (1) identify Lynch syndrome and (2) provide additional prognostic/predictive information in colon cancer Supported by scientific evidence	Scientific evidence Local data	• Expertise to perform testing • Policy pertaining to use (only to be used for certain indications) • Additional supplies (reagents)	Formal request/proposal to department; approved at departmental level Decision process lasted approx. 6 years with adoption occurring in 2012
Case 4: Barcoding	Technology in anatomic pathology to track cancer specimens from collection to reporting, and optimize patient safety Pre-post studies demonstrated significant error reduction	Scientific evidence Clinical experience Local data Data from other jurisdictions	• Capital equipment purchase • Education/training for all members of pathology team	Formal requests/proposals to successive levels of system, ending with government**; required approval at all levels Decision process lasted approx. 5 years with adoption occurring in 2014
Case 5: MRS	New staff position to optimize cancer patients’ access to non-intravenous prescription medications Limited scientific evidence to support innovation, through some descriptive data regarding institutional experiences in the US	Clinical experience Local data Data from other jurisdictions	• Social worker with expertise or willingness to develop expertise in medication access • Referral form/process • Evaluation framework and infrastructure	Ad hoc committee struck to address problem; recommendation approved at program level Decision process lasted approx. 2 years with adoption occurring in 2005

*PET* positron electron tomography, *IMRT* intensity-modulated radiation therapy, *MSI* microsatellite instability, *MRS* Medication Resource Specialist

*There were two options for accessing isotopes: purchasing from another province or making onsite with a cyclotron (which would require substantially more resources). Initially, isotopes were purchased from elsewhere, but a cyclotron was purchased and implemented approx. 4 years after PET implementation

**Required government approval because these innovations were large capital expenditures, requiring substantial funding

### Data collection

Data were gathered via two sources: documents and key informant interviews. Documents (e.g., policy reports, applications, planning documents, meeting minutes, advocacy letters, and evaluations) were acquired to gain historical and contextual perspectives on each case, to examine how scientific evidence was presented to and applied by those making the adoption decisions, and to corroborate and augment evidence from the interviews [19]. All documents were identified and accessed through key informants, Internet searches, and specific requests to individuals and organizations directly involved in the cases.

In-depth semi-structured interviews were conducted with key informants from each case to gain perspectives on and experiences with the decision to adopt the innovation. The research team purposively identified potential key informants based on their involvement in adoption efforts. This included decision “influencers” (e.g., clinicians advocating for the innovation) as well as decision “makers” [23]. Snowball sampling was also employed, with informants asked to suggest other individuals with whom it would be valuable to speak. An interview guide (see Additional file 1), with open-ended interview questions and related probes, was drafted based on the study objectives, team members’ relevant experiences, and CFIR [20], with practical guidance from Patton [24] and Rubin and Rubin [25]. CFIR was specifically used in the design of questions and probes to understand which factors influenced the decision-making processes. The questions were adapted based on each case as well as the person being interviewed and his/her role in the case.

XThree team members, all experienced in qualitative interviewing, conducted the interviews [CK for cases 1 and 2, RU for cases 3 and 4, and AF for case 5]. Interviews were carried out face-to-face or via telephone, depending upon practical considerations. All were audiotaped to ensure the data were captured and retrievable in true form, and transcribed verbatim by an experienced research coordinator. The interviewers met regularly to review the questions, ensure they were being answered in sufficient depth, and revise the questions or probes, as needed, before subsequent interviews.

### Data analysis

Detailed case descriptions were constructed for each case to describe its history, organization, and context. Data analysis involved an in-depth analysis of each case, followed by a cross-case analysis. First, the cases were treated as separate studies and analyzed independently using a thematic analysis approach [26], which involved iterative processes of coding, collating codes into categories, and generating, reviewing, and refining themes. To begin this work, team members [RU, CK, LLM] developed a codebook using both deductive and inductive concepts. LLM then used this codebook to code several transcripts, which resulted in the expansion and merging of existing codes through review and discussion with RU and CK. Upon agreement of codes, LLM then coded the transcripts and documents, one case at a time. The analysis was performed manually, with the assistance of qualitative software (NVivo) for data management and to facilitate comparison and synthesis of codes.

For each case, codes were collapsed into categories and larger themes through an iterative process that included critically analyzing each concept and category to identify similar and distinct concepts and categories; linking concepts and categories across all the data collected; reviewing the study objectives, conceptual framework (i.e., CFIR), and other literature sources; regular meetings with three team members [RU, CK, LLM]; and discussions with the full research team to facilitate questioning of the data and its analyses. The findings of each case were fully delineated before moving to the next case.

Next, a cross-case analysis was conducted to develop theoretically informed, generalizable knowledge on the role of scientific evidence in innovation adoption that may be applied to similar settings and contexts [19]. Multiple team discussions were held to permit the questioning, clarification, and confirmation of cross-case themes. This process included construction of tables to identify case-specific themes, understand how and the extent to which case-specific data were applicable across cases, and facilitate discussion of higher level themes related to the study objectives.

## Results

Across the five cases, data were collected from 32 key informants and > 100 documents. All interviews took place between December 2014 and April 2015. Key informants played differing roles in the cases, including advocating for the innovation, making formal requests for adoption/funding, and/or making the eventual adoption decision. They included clinical and administrative champions, departmental managers, department chiefs/heads, executive-level administrators (e.g., hospital VPs), policymakers from the provincial ministry of health, and community advocates. Due to the nature of their decision-making role, two key informants played roles in more than one case and were questioned about their role in each case.

The five selected cases are described in Table 1. Decision-making processes ranged from an informal approval process within a single department to formal approval at the departmental level to formal approval at various successive levels, including government. The timeframes for adoption also varied widely, from approximately 2 years to nearly a decade. Though not a sampling criterion, all innovations were proposed and advocated for by frontline staff, with none being top-down or externally mandated.

**Fig. 1:**
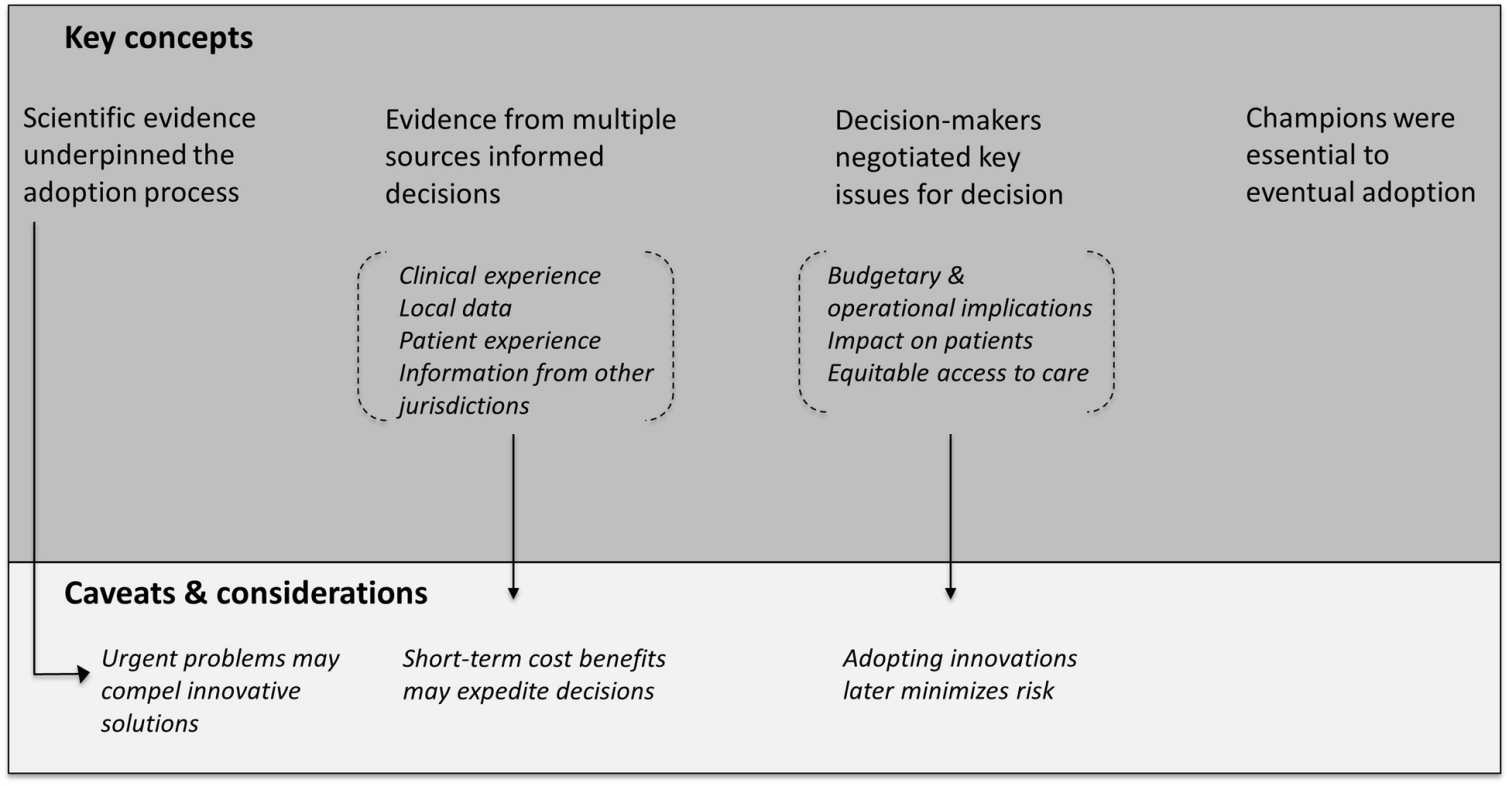
Key concepts and caveats and considerations in decision-making processes around adopting innovations in cancer care settings

The cross-case analysis revealed similarities across most cases. However, one case (case 5) fundamentally differed from the other cases in terms of the role of scientific evidence and the impetus for adoption. The findings are discussed below and broadly categorized into the “Key concepts” and “Caveats and considerations” sections. Figure 1 depicts these findings and their relationships.

### Key concepts

#### Scientific evidence underpinned the adoption process

The documentary and interview data revealed the adoption process played out on a continuum, from someone’s initial conviction the innovation should be implemented (typically someone at the frontline of care delivery), to advocating for the innovation (by an individual or small group of individuals at the frontline), to the decision to adopt the innovation at the departmental, organizational, and/or healthcare system levels. With the exception of one case (case 5), scientific evidence underpinned these processes. However, scientific evidence played a much greater role in early adoption processes when frontline personnel decided to advocate for the innovation. During subsequent decision-making (which occurred at the department, organization, and/or system levels), scientific evidence played a limited explicit role in decision-making processes, with decision-makers typically trusting the individuals who brought the innovation forward. As stated by one decision-maker involved in case 1:

We relied on the expertise of the Diagnostic Imaging professionals … I cannot speak to the evidence, I cannot point to the studies, except to say that it was confirmed with us, by both the hospital executive and DI, that there was ample evidence that this was the standard for technology. [P18]

A decision-maker in case 4 put it this way: “in a perfect world [where] we had lots of staff and lots of time … we’d probably have a more rigorous process and more data considered but … you know, we’d need a whole department for this” [P17]. In other words, at the formal approval levels, scientific evidence received only limited attention and consideration since most individuals at these levels felt the innovation would not be under consideration unless there was scientific evidence to support its adoption. In fact, application and proposal documents included limited information about the scientific basis for adoption. Rather, at these levels, both documentary and key informant data revealed issues related to capacity and costs dominated decision-making processes.

#### Evidence from multiple sources informed decision-making

In addition to scientific evidence, the data showed evidence from multiple sources informed decision-making, including (i) clinical experience, (ii) local data, (iii) patient experience, and (iv) information from other jurisdictions. Table 1 identifies the main sources of evidence by case. For example, in case 2, clinicians’ experiences with conventional radiotherapy and its associated toxicity and negative impact on quality of life reinforced the potential and direct benefit of intensity-modulated radiation therapy, a technology that, by design, provides more targeted delivery of radiation, thus minimizing exposure to surrounding tissues and critical structures. As one participant said, “the fact of the matter is that if you treat a certain organ to a lesser dose, there will be less side effects. It’s sort of self-evident, right?” [P4].

The use of local simulation data was also particularly useful in quantifying the advantage of this technology over conventional radiotherapy for the local patient population and thereby strengthening the support of local clinicians. Similarly, local data were key to influencing decision-makers to approve microsatellite instability (MSI) testing in Nova Scotia (case 3). Specifically, data demonstrating high accuracy and reproducibility of the test locally was paramount to the adoption decision. As one participant stated:

… to see whether we have this ability or capacity in Nova Scotia and to see whether the technique is really robust … it’s just showing local data to convince [the] committee you know, yes, [we] can do it. It can work here.” [P24]

Use of patient experience was explicit in case 1 only. Patient representatives, speaking at town hall meetings and with government officials and media, described the benefits of positron electron tomography (PET) and shared their experiences with having to access this technology outside the province. Their stories were well-articulated, emotive, and illustrative of the potential benefits of PET, helping to convince decision-makers and fundraising parties of the need for this technology in Nova Scotia. On speaking about the influence of patients, one participant said, “Even though [PET] had all these implications for research, you know, and … patient care and all these sorts of things … the part that really resonated with people was the story [patients] told” [P18].

Information from other jurisdictions was a particularly valued source of evidence for organizational-level decision-makers, providing them important insight into implementation challenges and real-world impact. This was especially pertinent to cases 2, 4, and 5. Whereas scientific evidence could provide insight into the *potential* impact for patients or the health system, understanding what had happened in other jurisdictions provided evidence into the implications and impacts outside of controlled research environments. As such, any scientific evidence that entered the decision-making process had to be negotiated alongside other sources of evidence, particularly as it provided limited guidance for action. One participant summarized the experience of scientific evidence and information from other jurisdictions by saying, “I think people prefer, you know, the highest level of research evidence possible. Uhm, but you know, there is great consideration given to what happens in other jurisdictions” [P17].

Related, the role of scientific evidence appeared less pertinent to adoption decisions when the innovation had been extensively adopted elsewhere, with the data suggesting real-world evidence and experience gained from others’ adoption was more highly valued.

#### Decision-makers considered three key issues when making decisions

Multiple sources of evidence sources were used given that decision-makers considered three key issues when making decisions: (i) expected budgetary and operational implications, (ii) expected impact on patients, and (iii) equitable access to care. These three issues prevailed regardless of what level the adoption decision occurred (i.e., departmental, organizational, or health system) though the different issues did not receive equivalent attention across cases or levels of decision-making.

Across all cases, cost considerations and financial pressures were pervasive. This was highlighted by a participant in case 3 when describing the approval process:

The last one was a big lab meeting… and we were kind of a little bit grilled. And, you know, what would this be and you are going to pay the cost and it was mostly about budget … So it was mostly people who were worried about dollars … it felt very much like, ‘tell us how this is not going to cost us anything and we might let you do it.’ [P27]

While cases 1 and 4 involved substantive capital expenditure costs, decision-making often involved consideration of other types of costs, including resource/training costs, ongoing operational costs, and opportunity costs. Regarding the latter, decision-makers continually remarked on the ubiquitous nature of competing priorities and recognized investing in an innovation will mean resources are not provided to other important programs, services, or innovations. This was summarized by one participant as:

Sometimes those big initiatives get juggled around from year to year because there’s just not enough money in the pot. And here’s the thing, there’s no unworthy things that come to our table. … There’s nothing that you can say that’s frivolous and we do not really need it. It’s unlimited need. So we are, we do ration, you know? [P14]

Another key budgetary and operational consideration related to maintaining scope. Specifically, decision-makers recognized that once an innovation is implemented, it is difficult to maintain boundaries around its use, particularly in a complex clinical care environment where evidence continues to emerge and evolve. This was true for cases 1–3, which were clinical (diagnostic and therapeutic) innovations. As stated by one participant in case 2, “We couldn’t open the floodgates. We couldn’t overload the system with a whole range of indications that could be treated, even though many, it seemed at that time, would benefit from this technology” [P1]. Thus, policies were put in place to ensure use of the innovations would be limited to specific indications and referring clinicians to contain costs and resources once implemented.

The second key issue was expected patient impact, that is, expectations about the nature and magnitude of patient impact were important criteria when considering a particular innovation given competing priorities. Expectations of patient impact were informed by a variety of sources, including the scientific evidence base and information from other jurisdictions. For example, one participant from case 2 noted the tremendous impact IMRT has on patient quality of life and how this influenced adoption:

When a patient gets xerostomia, it’s terrible. They have to carry around a water bottle for the rest of their lives because the damage is permanent, and pretty much every nasopharyngeal patient had that side effect prior to this technology … So there was some very compelling data … and the answer was that this side effect was virtually gone. [P1]

Another participant more generally noted, “there has to be a benefit somehow to patient care. The cost is something, probably the next thing we look at” [P10].

The third issue in the decision-making process was whether or not a particular technology or service was standard of care elsewhere—specifically, equitable access to care was viewed as an important value, and if other Canadians had access to a particular technology/service, then patients in Nova Scotia should as well. Those advocating for the innovation and its adoption often presented this equity argument, highlighted by the following participants from cases 1 and 2, respectively:

The fact that we didn’t have [PET] in Atlantic Canada was also a great motivation to bring it here because it said that the standards of care … were better frankly, everywhere else in Canada except here. [P18]

Our argument was always like ‘we can do it here, [so] why can we not do it here?’ Why should patients have to travel all that way to Toronto? They do not want to travel. Who wants to travel out of province for a treatment? And so we said ‘well look, if we don’t do this now, we’re always going to be sending people out of province, and what good is that?’ [P4]

At the same time, decision-makers at the organizational and system levels described reconciling their goal of equitable access with real-world cost and resource considerations as well as expected patient impact (relative to alternatives and other innovations).

#### Champions were essential to eventual adoption

Across all five cases, local champions, who were dedicated to the innovation and promoted it tirelessly within their departments and organizations, were critical to eventual adoption. In cases 1–3, it was the mounting scientific evidence that influenced these individuals to become champions for the innovations. Champions were instrumental in motivating others to support the innovation and overcoming barriers to its adoption and eventual implementation. These individuals undertook a number of activities, including gathering and disseminating multiple types of evidence to communicate benefits, formally and informally advocating for the innovation across levels of the organization/system, collecting local data to demonstrate need and/or value, and lobbying with external players (e.g., hospital foundations, policymakers, politicians) to garner moral and material support. For cases wherein barriers were substantial (e.g., case 1), the champion played a larger role in selling and moving the idea forward. As one participant stated, “he is the driving force behind it … The reason the PET machine is here is because of [him]. He was the champion for it and he wouldn’t take no for an answer. He made it happen” [P18]. In fact, the data suggested even in cases were the evidentiary basis and existing infrastructure were robust, adoption would not have happened without a champion continually pushing the innovation forward.

### Caveats and considerations

#### Urgent problems may compel innovative solutions

Case 5 differed in many respects from the other four cases. In particular, the innovation (Medication Resource Specialist; MRS) was implemented due to a perceived need to address an urgent problem at the local level. Specifically, the organizational (constrained human health resources, increasing volume of cancer patients seen) and socio-political (high proportion of population with no or limited insurance coverage, rapid emergence of high cost drugs, changing treatment landscape not reflected in the provincial drug formulary) context at the time resulted in an urgency to *do something* about medication access within the cancer center. At the same time, frontline staff (social workers in particular) felt unable to effectively navigate the increasingly complex environment of medication access and to adequately meet the high psychosocial needs of their patients because of the large amount of time they spent on medication access issues.

So it was hugely time consuming … and it was also difficult to keep in your head all of the ins and outs and the complexities of all of these medications … we did not always have the capacity to be able to jump through all the hoops quickly enough because there were so many needs … So more and more and more time was being spent on medication resource issues and less and less time was being spent on practicing scope. [P28]

In fact, documentary data demonstrated that, in 2003, social workers spent more than 30% of their time coordinating medication access and funding.

While the need was informed by various sources of evidence (clinical experience and local survey data), there was limited scientific evidence available to inform the development of the resulting innovation. This was expressed by one participant as:

In terms of this particular medication resource specialist, as far as my understanding was, there was no actual evidence to support that it was going to help us … it was just such an obvious need you didn’t need any evidence. [P29]

Thus, the data suggested the differences inherent in this case were due to the nature of the problem, and not necessarily the nature of the innovation. Development of the MRS position was viewed as a creative solution to a substantial local need, and one that could generate evidence versus being informed by evidence.

#### Short-term financial pressures may expedite decisions

Case 5 was also unique in that the solution was addressing a cost and resource problem, with administrators anticipating substantive cost benefit in the short term. Specifically, as cancer center staff felt unable to effectively navigate the complex environment of medication access and coverage, they increasingly relied on the hospital’s No Insurance (NOINS) program, which covered urgent medication costs for patients when all other options were exhausted. One participant described the perception that NOINS “was being overused, not because people thought it was easier or an easy way out, it was purely because people didn’t know what else to do” [P31]. As these costs escalated, a solution to medication assistance became critical. Many participants perceived this cost crisis as central to administrators’ support for the innovation and its expediency into practice. Unlike the other cases, the MRS position was directly solving a problem for administrators themselves in addition to a clinician and patient problem. Indeed, administrators were highly involved in and supportive of the innovation and allowed it to evolve over time as needed, as noted by one participant who stated: “we had good support from our senior leader” [P32]. Documentary evidence demonstrated a 47% reduction in NOINS costs over the first 3 years of the position.

#### Adopting later minimizes risk

For cases 1–4, data pertaining to decision-making at the organizational and/or health system levels uncovered the wide-ranging view that adopting innovations after they have been adopted and implemented elsewhere had discernible advantages. Specifically, adopting later in time (relative to peer institutions elsewhere) minimized risk and allowed managers/administrators to acquire valuable evidence from elsewhere to understand the resource implications and real-world patient/health system impacts of implementation. This evidence could inform adoption and implementation efforts and lower any ambiguity associated with the innovation. This was described by one participant who said, “Yes, it’s new, it’s great, it’s wonderful. But we’re not ready for that. And let’s let some other institution get the bugs out and then we’ll go forward” [P11].

Participants across these four cases described an implementation cascade where innovations were developed and implemented elsewhere, and eventually trickled down to their organization. While this cascade had ostensible benefits for decision-makers, frontline clinicians were continually frustrated by what they saw as a lack of innovation and improvement in their respective settings.

## Discussion

This study sought to better understand the role of scientific evidence in adoption decisions. We found scientific evidence typically underpinned the adoption process (with the exception of one case), yet had a declining role in decision-making as the decision progressed to high levels of the organization and system. We also found that decision-makers often relied on multiple types of evidence, which were necessary due to the multiple issues they considered as they made their decisions. The most relevant type of evidence for organizational-level decision-makers was information from other jurisdictions that had previously implemented the innovation since this evidence provided them with key insights into implementation challenges and real-world impact. These findings contribute to recognized gaps in the literature base, including ascertaining how and why different types of evidence are used during decisions to adopt innovations in health care [15, 16].

Research has shown that the strength or quality of scientific evidence does not always have a large influence on the decision to adopt innovations in health care [27, 28]. For many decision-makers, experiential knowledge can feel more relevant and applicable than knowledge acquired through scientific inquiry [29, 30]. A 2017 scoping review to map the literature on how evidence informs decisions to introduce innovations in health care settings found that, although scientific evidence was the most reported type of evidence used during decision-making, three-quarters of the included studies demonstrated use of non-scientific forms of evidence, including local data and clinical/professional experience [15]. In this study, the value attributed to a particular type of evidence differed across levels of the organization, with scientific evidence highly valued by frontline clinicians (mainly specialist physicians) who were advocating for the innovation, whereas non-scientific types of evidence were highly valued by the individuals making the adoption decisions (e.g., department/unit chiefs and managers, senior executives, policymakers). The latter included patient stories (one case only), local data, and data from other jurisdictions. This reliance on multiple types of evidence may be expected given that, on any single issue, decision-makers must balance clinical effectiveness and need with budgetary, capacity, patient/public, and other considerations [31, 32]. Others have also found that the earlier adoption stages focus on assessments of efficacy and safety, with later stages focusing on issues related to implementation (e.g., acceptance, ease of use) [33, 34]. This shift from assessing an innovation’s scientific merit to understanding its “complete value” underscores the need to consider multiple types of evidence as well as important contextual factors [35].

In four cases, decision-makers used local data to inform their decisions on whether or not to adopt the innovation. While the nature of the local data varied (e.g., simulation, service provision/resource use, serious events, patient satisfaction), the findings suggested the desire for local data was twofold: it allowed decision-makers to better understand the nature and scope of the problem in their setting and/or enabled them to better estimate the local impact on their patient population. Indeed, when deciding to adopt complex innovations, many of which are resource-intensive, decision-makers must negotiate the knowledge base (e.g., reported clinical effectiveness) with local needs (e.g., nature/extent of the problem in their setting/jurisdiction). The proclivity for local data aligns with a recent study on policymakers’ uses and preferences for evidence in public health whereby policymakers reported local data as their most highly valued type of evidence [12].

Organizational decision-makers placed especially high value on information they acquired from other jurisdictions. This information was particularly germane to evaluating the potential risks of adoption in that it provided real-world experiences about the budgetary, operational, and patient impacts of the innovation, as well as implementation challenges and successes. Thus, while such evidence may be used to understand the real-world benefits of an innovation, it may also be used to mitigate potential risks to the organization. Decision-makers across cases discussed acquiring such information from colleagues in other jurisdictions, gray literature sources, industry/vendors, and/or networking at meetings and conferences. Recent studies have shown that policymakers in public health report their most frequent source of evidence as “other people” (e.g., colleagues) [12] and service payers in healthcare systems favor the evidence they receive from contact with colleagues or through professional networking over scientific evidence [15]. A study on technology adoption and implementation in nine UK acute care organizations found that managers tend to prioritize implementation and cost evidence from within their own or other hospital organizations over scientific evidence [35]. Similar to our study, managers also noted that evidence generated from research failed to provide direction or prescriptions in terms of taking action. Recognizing that varied types of evidence are sought and evaluated for policy decisions in health care, Oliver and Pearce [16] recommend considering the kinds of questions policymakers ask and how this might affect the type of evidence used. They suggest typical questions might be “what is the nature of this problem?” and “what have others done?”—both of which require types of evidence that may be unrecognized or underappreciated within today’s accepted hierarchies of evidence [16].

The enabling influence of on-the-ground champions cannot be understated. Across all cases, the presence and role of champions was paramount to decisions to adopt the innovations. The importance of champions in adoption and implementation processes has been demonstrated in many studies [36–40] and is incorporated into relevant models/frameworks [41–43], including CFIR [20]. A recent systematic review on the organizational factors influencing the implementation of evidence-based practices found the presence of champions was important in approximately one-third of the included studies [44]. Given their enabling influence, those leading adoption and implementation efforts must consider how best to support (morally and materially) these individuals in their roles, particularly as championing is typically done as an add-on to one’s clinical or managerial role, and what will happen should the champion leave the role (e.g., retire, change positions, or simply burn out).

Case 5 differed from the other cases in many respects. Certainly, the nature of this innovation (e.g., a new human resource) was quite different than the other innovations (e.g., new technologies). At the same time, this case may be characterized as a problem-based initiative wherein a solution was sought to a perceived urgent problem, whereas the other cases may be characterized more as solution-based initiatives with clinicians advocating for specific solutions (innovations) they perceived would benefit patient care, safety, or outcomes in their respective areas. Moreover, the data suggested the differences inherent in this case were due to the nature of the problem and not necessarily the nature of the innovation, that is, senior administrators were keenly aware of the high, and ostensibly avoidable, cost and resource burden of the existing way of accessing medications—and reducing these costs became a clear priority of these organizational leaders. Indeed, innovations are more likely to be adopted when it aligns with the organizational needs and addresses clear, practical problems [15]. Critical events and pressures can also have significant impacts on evidence use during the decision-making process, often resulting in a less rigorous approach to evidence use and an adoption of innovations with low or emerging evidence of efficacy [35].

There are several practical implications of these findings in terms of informing adoption efforts. One relates to the value of information on real-world implementation and impacts. Given that decision-makers must always examine the potential benefits and costs (e.g., potential negative impacts) of an innovation [18], real-world evidence is imperative to understanding both of these issues. A second is that the adoption of complex innovations typically involves a number of stakeholders, in varying professional roles and at different levels of the health system. As a consequence, it is critical that the evidence used to support adoption is relevant to and addresses the interests or concerns of all these stakeholders. In reality, this will only be achieved via the use of multiple sources and types of evidence. A third implication is the influence of/reliance on champions to advocate for and push innovations through the various levels of the adoption decision. Although champions often emerge as a key determinant of adoption and implementation, as others [41] have pointed out, there is no simple recipe for how champions should act that is independent of the innovation or the context in which the change is being considered.

This study has a number of limitations. First, this study was conducted in one jurisdiction only, which may limit the applicability of findings to other settings. However, our sampling strategy ensured the cases varied on a number of key features (including contextual aspects) and thus sought to optimize transferability across types of innovations and contexts. In addition, healthcare systems generally have a number of defining features regardless of their location, including wide-ranging and diverse stakeholders and complex governance arrangements [45]. Both should increase the applicability of these findings outside the province of study. Moreover, this study provides rich information for learning purposes and may serve to elaborate on or reformulate existing theory (i.e., analytic generalization), an important outcome of case study research and qualitative inquiry in general [22, 46].

Second, in each case, some individuals were difficult to reach because they had changed positions or retired since the innovation was adopted and implemented. As a result of not reaching certain individuals, we may have missed some important data. Similarly, given that the time period between adoption and interviews was lengthy for several cases (particularly cases 2 and 5; see Table 1) the data are subject to issues of recall. However, key informants’ recollections of people, events, and processes during the adoption efforts did not differ substantively from one another and were triangulated by documentary evidence. Third, although not a sampling criterion, all of the innovations studied were driven by frontline staff. Thus, the findings may be more applicable to such initiatives versus those driven by senior leadership or external organizations. Finally, given that we purposely did not select cases where the innovation was not adopted, we cannot comment on the role of scientific evidence in this setting.

There are also a number of study strengths. We used several methods to collect data, with the documentary data supporting and confirming the data we gathered from key informant interviews. We also undertook a number of steps to optimize rigor, including systematic coding with regular meetings to review and discuss coding, and multiple team discussions to question, clarify, and confirm the case-specific and cross-case themes. Team members had expertise in case study methods, clinical care/service delivery (surgery, oncology, diagnostic imaging, pathology), and health administration, ensuring the data were viewed and questioned through multi-disciplinary perspectives. As such, this study contributes important data to help address identified gaps in the literature on how evidence is used in decisions to adopt innovations in health care organizations.

## Conclusion

This study demonstrated how research evidence is considered among other sources of evidence as decisions are made to adopt innovations in health care. The findings revealed the different types of issues decision-makers consider while making these decisions and why different sources of evidence are needed in these processes. Future research should continue to examine how evidence is used in adoption decisions, including how different types of evidence are legitimized and why some types are prioritized over others. Given the high value placed on information from other jurisdictions, a better understanding of how this evidence is constructed and applied during decision-making processes is also warranted.

## Additional file

Additional file 1: Draft Interview Guide: Clinician or administrator involved in the adoption process. (DOC 29 kb)

## Abbreviations

CFIR: Consolidated Framework for Implementation Research
IMRT: Intensity-modulated radiation therapy
MRS: Medication Resource Specialist
MSI: Microsatellite instability
PET: Positron electron tomography
