# Supplementary material for: The role of scientific evidence in decisions to adopt complex innovations in cancer care settings: a multiple case study in Nova Scotia, Canada
Source: Implement Sci. 2019 Feb 12;14:14. doi: 10.1186/s13012-019-0859-5 (PMC6371509; doi:10.1186/s13012-019-0859-5)
Supplement: Supplementary file 1 — Draft Interview Guide: Clinician or administrator involved in the adoption process. (DOC 29 kb) [file 13012_2019_859_MOESM1_ESM.doc]

**Draft Interview Guide: Clinician or administrator involved in the adoption process.**

The draft interview guide was revised over time and adapted based on the specific case and individual interviewed. The ongoing analysis of key documents also resulted in modification and fine-tuning of questions relevant to each case.

1. Tell me about your experience with the adoption of [*Innovation X*] in Nova Scotia.

What was your specific role?

What did you believe would be the main benefits of adopting it?

1. Take me back to how [*Innovation X*] was introduced here.

Who brought the proposal or application forward?

Why did he/she/they propose it?

How was it presented (presentations, workshops, etc.)? How was it perceived?

What information was included in the proposal/application?

How was this information considered and dealt with?

Did anyone challenge the proposal/information presented? If so, how so?

1. How did the decision-making process unfold?

What form did the decision-making process take? How long did it take?

Who was involved?

Were there disagreements? [*If yes, ask to describe*]

Were there areas of obvious agreement? [*If yes, ask to describe*]

1. How did you view the quality and strength of the research evidence that was available?

Were there differences in terms of how people viewed this evidence?

How did this evidence feature in the decision-making process?

Was this evidence sufficient to make your decisions? Why or why not?

1. What other types of evidence did the decision-making team feel it needed in order to make an informed decision about [*Innovation X*], if any? Probes: local evaluation data, cost-effectiveness data, patient/public views

Why was this information needed?

How was it obtained?

How was it considered and dealt with?

Was any of the information controversial or contentious in any way? If so, how so?

Out of these “sources of evidence”, which do you perceive were most important to the decision?

Did a hierarchy of evidence emerge when considering these different types?

1. What other things played a role in deciding to adopt [*Innovation X*]? How so?

How would you characterize each of these things?

How were they considered/managed amongst the decision-making team? Amongst other relevant stakeholders?

*Probes*

Innovation: relative advantage over existing tools/alternative solutions, degree to which it could be adapted/refined to meet local needs, perceived difficulty of implementation, costs of tool + its implementation

Inner setting: degree to which people saw a need for innovation, relative priority compared to other initiatives, degree of fit with departmental/organizational interests and priorities, degree of fit with existing workflows and systems, perceived (dis)incentives, available resources (e.g., funds, skilled staff, physical space), degree of commitment (from senior management, frontline staff)

Outer setting: policy and regulations, political environment, external recommendations/ guidelines, patient needs, public values, capacity/resource issues, pressing/competing priorities

Process: relationship to/trust in the individuals proposing/endorsing the innovation, degree to which an implementation plan was developed, engagement of appropriate individuals (e.g., credible colleagues)

1. To what extent did people have to be persuaded to adopt [*Innovation X*]? How did this occur?
2. Who made the final decision to adopt? Are you able to say?
3. Thinking through everything we have talked about, from your perspective, what sources of evidence were really necessary to making the decision to adopt [*Innovation X*]?
4. Are there any other topics related to the adoption of [*Innovation X*] you would like to comment on?
